# Reflection on osteoarticular infections in children

**DOI:** 10.3389/fped.2023.1280878

**Published:** 2023-10-27

**Authors:** Giacomo De Marco, Blaise Cochard, Giorgio Di Laura Frattura, Silvia Valisena, Ludmilla Bazin, Dimitri Ceroni

**Affiliations:** ^1^ Pediatric Orthopedics Unit, Pediatric Surgery Service, Geneva University Hospitals, Geneva, Switzerland; ^2^ Division of Orthopedics and Trauma Surgery, Geneva University Hospitals, Geneva, Switzerland

**Keywords:** infection, osteoarticular, pediatric, *Kingella kingae*, NAAAs

The management of osteoarticular infections (OAIs) has changed drastically over the past 20 years thanks to the advent and widespread use of nucleic acid amplification assays (NAAAs). Molecular techniques have substantially improved the detection of low levels of bacteriological agents in clinical samples and have decreased the rate of “culture-negative” OAIs (1, 2). A better understanding of the pathophysiological mechanisms involved in infections has radically changed our understanding of the etiology of OAIs (3–5), resulting in major changes in diagnostic approaches and therapeutic management.

Since then, concepts about the etiology of OAIs have been reviewed and improved. They now highlight that *Kingella kingae* must be considered one of the main pathogens responsible for the hematogenous infection of bones, joints, intervertebral discs, and tendon sheaths, especially in children aged between 6 and 48 months old. *K. kingae*'s role in OAIs was underestimated in the past (3–6), but growing evidence has downsized the role of *Staphylococcus aureus*, considered the predominant pathogen responsible for OAIs in the pediatric population for many decades (6–17).

More accurate identification of the bacterial pathogens responsible for OAIs has also led to the development of new concepts of care for pediatric populations. It is now recognized that the clinical and biological aspects of OAIs are closely related to a child's age and, above all, the pathogen responsible. We now know that there are different bacteriological specificities for the OAIs in different age groups of children and teenagers. Other factors, such as a patient's socioeconomic status, potential comorbidities, vaccination status, changes in patterns of immunomodulating diseases, and the emergence of resistant bacteria, also seem to play significant roles in OAIs and must be considered (18). Today we realize how important it is to consider all the patient's characteristics to fully appreciate an OAIs clinical history.

Despite the publication of guidelines on diagnosis and management of OAIs in pediatrics (19), there is still no real worldwide consensus on the treatment of these infections in children. Nevertheless, pediatricians and pediatric orthopedists do agree that OAIs clinical presentation and the subsequent clinical outcome will differ depending on its microbiological causes, and, therefore, the treatments required may differ substantially from patient to patient. For example, OAIs caused by pyogenic pathogens, such as such as *S. aureus*, *Streptococcus pyogenes*, *Haemophilus influenzae* type b or *Streptococcus pneumoniae*, usually present with more severe symptoms and clinical manifestations, show a slower clinical response, and have potentially worse outcomes. These OAIs may thus require invasive diagnostic and therapeutic procedures and lead to intravenous antibiotic treatments (20, 21). Conversely, the OAIs due to *K. kingae* usually characterize by lesser general and local inflammatory reactions no long-term orthopedic sequelae, and are accessible to antibiotic therapy alone (3), without surgery.

The initial and historical controversy between pediatricians and pediatric orthopedists, about the indications for surgery in these infections, withered with better knowledge about the clinical manifestations of infections depending on the causative pathogen. There is now general agreement that certain OAIs—those due to fastidious bacteria, such as *K. kingae* or *Moraxella* spp.—can be treated using intravenous or even oral antibiotics alone, with no need for surgery (22). Severe infections, however, still require surgical management alongside intravenous antibiotic therapy, especially if pathogens such as *S. aureus* or *S. pneumoniae* are involved.

Thanks to this new approach, OAIs can now be classified into two groups: those which constitute genuine orthopedic emergencies that usually require surgical treatment alongside antibiotics therapy, and those which appear much less serious and can be treated with oral or intravenous antibiotics and without surgery. Making this distinction seems essential since it will probably significantly change the standards of care for pediatric OAIs in the future. It is even more crucial since the notion of what constitutes an emergency in cases of OAIs is not the same for pyogenic and non-pyogenic pathogens. A rapid identification of the responsible pathogen and a proper clinical and radiological diagnosis are crucial to avoid any serious complications.

To adapt a treatment to a specific clinical situation, two fundamental conditions must be met. First, the responsible pathogen must be identified as quickly as possible, and its susceptibility to selected antibiotics determined. Second, targeted clinical radiological investigations, such as x-rays and magnetic resonance imaging (MRI), should be used as crucial, reliable tools to help decision-making processes for surgical procedures. Treating OAIs with close regard to the pathogens and the local impact of the infection also depends on the real-time availability of the above-mentioned instruments in the patient's medical facility.

Regarding the identification of the pathogens responsible for OAIs, NAAAs technology has progressed significantly in the last few years, and improvements have been recorded in efficiency (the development of primers amplifying species-specific targets better than universal primers) and specificity (assays less prone to contamination) (1, 2). With the latest polymerase chain reaction processes, it is now possible to generate millions of copies of a specific DNA sequence in just a few hours. We could expect there to be continuous improvements in nucleic acid amplification processes, probably leading to faster bacterial identification and eventually to real-time diagnosis. This was the case of *K. kingae*, whose identification was dramatically improved using NAAAs (23).

In addition to their ability to identify pathogens, some NAAAs can detect the presence of genes encoding proteins such as antibiotic-inactivating enzymes or virulence factors (24). With the continuous improvement of NAAAs technologies, we imagine that these could potentially replace the use of standard cultures for antimicrobial susceptibility testing (24). Finally, it is legitimate to think that next-generation metagenomic sequencing of microbial cell-free DNA may become, in a close future, an attractive diagnostic modality for the infectious diseases, allowing rapid broad-range pathogen detection by means of noninvasive sampling such as peripheral blood (the so-called liquid biopsy) (25).

Regarding radiological explorations, MRI is currently the best diagnostic instrument for accurate OAIs investigations and workups. It plays a central role in current clinical algorithms (26, 27), providing fast, reliable diagnoses and helping orthopedic surgeons in their decision-making processes, especially when a decision on surgery must be taken. When acute OAIs is suspected in a child, an emergency MRI aims to identify bone marrow signal abnormalities and joint effusions, offering substantial benefits in pre-operative planning (27–29). However, MRI is not always readily available or accessible, and the procedure's duration can constitute a problem, especially in young children, because the patient must maintain the same position for a long time. Using lower-resolution data acquisition and compressed reconstruction could reduce total scan time and costs.

The next few years will certainly be marked by a revolution in the management of OAIs, both in the diagnostic approaches used and in their treatment. In the very near future, the rapid identification of the causative pathogen, the determination of its sensitivity to antibiotics, and a precise definition of its location will enable pediatricians and surgeons to provide patients with more specifically targeted treatments. It is now up to primary care physicians and specialists to keep up to date on the progress made in the fields of bacteriological and radiological investigation and then provide their patients with the best possible treatments.
